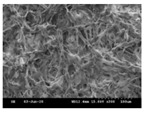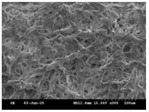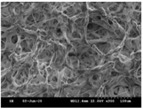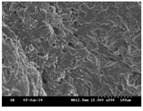# Development of Active Packaging to Extend the Shelf Life of *Agaricus bisporus* by Using Plasma Technology

**DOI:** 10.3390/polym13132120

**Published:** 2021-06-28

**Authors:** Chao-Kai Chang, Kuan-Chen Cheng, Chih-Yao Hou, Yi-Shan Wu, Chang-Wei Hsieh

**Affiliations:** 1 College of Biotechnology and Bioresources, Da-Yeh University, 168 University Rd., Dacun, Changhua 51500, Taiwan; kai0913077636@gmail.com; 2 Graduate Institute of Food Science and Technology, National Taiwan University, 1, Sec 4, Roosevelt Road, Taipei 10617, Taiwan; kccheng@ntu.edu.tw; 3 Institute of Biotechnology, National Taiwan University, 1, Sec 4, Roosevelt Road, Taipei 10617, Taiwan; 4 Department of Medical Research, China Medical University Hospital, Taichung 40400, Taiwan; 5 Department of Optometry, Asia University, 500, Lioufeng Rd., Wufeng, Taichung 41354, Taiwan; 6 Department of Seafood Science, National Kaohsiung University of Science and Technology, 142, Haizhuan Rd., Nanzi Dist., Kaohsiung City 81157, Taiwan; chihyaohou@gmail.com; 7 Department of Food Science and Biotechnology, National Chung Hsing University, 145 Xingda Rd., South Dist., Taichung 40227, Taiwan; elle5130675@gmail.com

**Keywords:** plasma modification, preservation, carboxymethyl cellulose, collagen, *Agaricus bisporus*

## Abstract

In this study, a preservation package that can extend the shelf life of *Agaricus bisporus* was developed using plasma modification combined with low-density polyethylene (LDPE), collagen (COL), and carboxymethyl cellulose (CMC). Out results showed that the selectivity of LDPE to gas can be controlled by plasma modification combined with coating of different concentrations of CMC and COL. Packaging test results applied to *A. bisporus* showed that 3% and 5% of CMC and COL did not significantly inhibit polyphenol oxidase and β-1,3-glucanase, indicating no significant effect on structural integrity and oxidative browning. The use of 0.5% and 1.0% CMC and COL can effectively inhibit the polyphenol oxidase and β-1,3-glucanase activity of *A. bisporus*, leading to improved effects in browning inhibition and structural integrity maintenance. P-1.0COL can effectively maintain gas composition in the package (carbon dioxide: 10–15% and oxygen: 8–15%) and catalase activity during storage, thereby reducing the oxidative damage caused by respiration of *A. bisporus.* The current study confirmed that the use of plasma modification technology combined with 1.0% COL can be used in preservation packaging by regulating the respiration of *A. bisporus*, thus extending its shelf life from 7 to 21 days.

## 1. Introduction

White mushrooms (*Agaricus bisporus*, *A. bisporus*) are among the most popular and common edible mushrooms because of their functional properties. They are characterized by good texture, high nutritional value, unique flavor, and high-protein and low-fat contents compared with other vegetables [1,2]. Due to their high respiration rate, high moisture content, and the absence of cuticle after harvesting, rapid senescence, browning, deterioration, and microbial contamination is observed.

Respiration is a series of reactions that accelerate the decomposition of tissues after the mushroom leaves the culture medium to maintain its own physiological metabolism, which includes the degradation of cell walls and the production of reactive oxygen species. The mushroom cell wall, which is composed of chitin and β-glucan, is gradually decomposed by enzymes; the firmness is rapidly reduced and tissue autolysis causes accelerated deterioration [2,3]. Reactive oxygen as well as free radicals produced by respiration accelerate the oxidation of phospholipids on cell membranes, resulting in the production of peroxide malondialdehyde (MDA) and leakage of electrolytes in cell membranes. Furthermore, polyphenol oxidase further consumes the phenolic compounds in the mushroom and causes oxidative browning [4,5,6]. Although the mushroom is stimulated by high oxygen and its internal antioxidant system, including superoxide dismutase, ascorbate peroxidase, and catalase (CAT), as well as other activities that temporarily increase the mushroom’s hydrogen peroxide conversion to O_2_ and H_2_O in plants [7], the vigorous respiration still causes the activity of the antioxidant system to decrease.

Modified atmosphere packaging (MAP) is the most common packaging technology and it is mainly achieved by replacing and filling different partial pressure gas components (oxygen, carbon dioxide, and nitrogen) to create a gas balance in the package. Currently, the most common MAP practice is to reduce the oxygen concentration to <1% [8,9]. However, most edible mushrooms such as *A. bisporus* perform extensive respiration after harvest and oxygen in the package gets rapidly consumed. The low oxygen environment (oxygen concentration of <1%) then promotes anaerobic respiration, leading to amino acid consumption for energy source as well as production of ammonia and other metabolites, resulting in serious deterioration of the smell, appearance, and structure of the mushroom [10,11]. Even if the partial pressure of oxygen, carbon dioxide, and nitrogen suitable for mushroom preservation is initially adjusted, the respiration of mushroom disrupts this gas balance and affects its preservation ability. Equilibrium MAP (EMAP) is primarily used for fruits and vegetables. No additional gas filling is required and the gas environment in the packaging is dynamically adjusted mainly by the selection rate of the packaging film for oxygen and carbon dioxide. However, traditional packaging or modified air packaging cannot adapt to the dynamic changes in plant respiration. Therefore, in recent years, research on preservation packaging has gradually focused on EMAP [12].

Traditional mushroom packaging such as low-density polyethylene (LDPE) and polyvinyl chloride (PVC) films have been previously used in several applications in food packaging [13]. However, the lack of gas selection of these films and the relatively low water vapor transmission rate [14,15] make them inappropriate for modifying the atmospheric concentration of oxygen and carbon dioxide in packaging aimed at extending the shelf life. Gholami et al. [16] mixed polyethylene terephthalate (PET), polysaccharides, and linear low-density polyethylene (LLDPE) with polymer materials that have different selectivity for gases to build a gas selective membrane that extends the shelf life of *A. bisporus* from 7 to 10 days. Nevertheless, when the gas barrier effect of the package is high, the *A. bisporus* in the package starts to deteriorate, owing to anaerobic respiration after 14 days. Although this study exhibited the effect of regulating the respiration of *A. bisporus* by coating different materials, it did not provide insights on controlling the concentration of coating material on the respiration of *A. bisporus*. Currently, research on food preservation with natural polymers is one of the important research projects in food science [17]. Collagen (COL) and carboxymethyl cellulose (CMC) are common food raw materials and also a kind of macromolecular polymer used in related research and applied in the preservation of edible film [18]. Previous relative research has revealed that COL and CMC have gas selectivity toward carbon dioxide, oxygen, and nitrogen [19,20,21]. However, the opacity and lack of ductility of COL and CMC are factors restricting the use of COL and CM in the development and application in EMAP packaging [22,23,24].

Plasma modification technology is widely used in material modification. Its main mechanism is to activate the surface of the functional groups of the material by the activation energy of the plasma to further increase the amount of functional groups on the polymer surface [25,26]. Our previous research showed that when LDPE is modified by plasma, it is more advantageous to coat CMC, COL, and cinnamaldehyde on LDPE and create better tilapia preservation effect [27,28]. This finding proved that plasma modification could retain COL and CMC on traditional mushroom packaging materials such as LDPE to regulate the gas composition in packaging.

Relevant studies have confirmed that active multilayer films have been used for controlled release [29,30] and to improve the antioxidant capacity and antibacterial capacity [31] as well as the physical structure and other properties of packaging material [32], which indicates that the combination of composite polymers used in the development of food packaging is an important trend in polymer research.

On the basis of the above, this study aimed to use plasma modification technology by casting COL and CMC on the plasma-treated LDPE surface as well as to explore the utility of packaging films that exhibit appropriate gas permeability to determine whether the COL- and CMC-coated films demonstrating suitable gas permeability can extend the shelf life of *A. bisporus* during storage period.

## 2. Materials and Methods

### 2.1. Materials

*A. bisporus* was bought from the Hecheng Commercial Firm (Taichung, Taiwan) and stored at 4 °C. *A. bisporus* was chosen on the basis of size uniformity, color, and it having not had any mechanical damage or disease to ensure the experiment’s accuracy. COL was obtained from Asahi Gelatine Industrial CO., LTD (Hyogo, Japan). CMC was obtained from ShowaKako corporation (Osaka, Japan). LDPE was obtained from Chinn Hui Co., Ltd. (Tainan, Taiwan) Ethanol (95%) was obtained from Summi Instruments Co., Ltd. (Changhua, Taiwan). Calcium chloride dehydrate (>95%), 99.5% D-(+)-glucose, 80% glucan, 99% catechol, 98% 3,5-dinitrosalicylic acid (DNS), and 99% citric acid were obtained from Sigma-Aldrich (St. Louis, MO, USA). Thiobarbituric acid (98%) was obtained from Alfa Aesar (Haverhill, MA, USA). Hydrogen peroxide (30%), sodium hydrogen phosphate (99%), sodium dihydrogen phosphate (98%), sodium citrate (96%), sodium hydroxide, and potassium hydroxide (>85%) were obtained from Katayama Chemical Co., Ltd. (Osaka, Japan). Trichloroacetic acid (>99%) and potassium sodium tartrate tetrahydrate (99%) were obtained from DaeJung Chmicals and Metals (Gyeonggi province, South Korea). Deoxidizing agent was obtained from Taiwan Dorency Co., Ltd. (Taichung, Taiwan).

### 2.2. Preparation of Composite Films and Package

Plasma treatment was performed as previously described by us, with slight modifications [27]. LDPE films were cut in pieces of 9 × 7 cm^2^, cleaned with alcohol to remove dust or oily compounds from the film’s surface, and dried before plasma treatment. The LDPE films were treated in a plasma reactor using radiofrequency plasma (13.56 MHz) that was fitted with a capacitively coupled parallel-electrode system with an automatic matching device (Junsun Tech Co., Ltd., Hsin-Chuang, Taiwan). Films were treated with 30 W for 60 s and immediately coated with COL and CMC solutions (0.5, 1.0, 3.0, and 5.0% wt %) separately and were then dried at 25 °C for 24 h. These films were marked as P-0.5COL, P-1.0COL, P-3.0COL, P-5.0COL, P-0.5CMC, P-1.0CMC, P-3.0CMC, P-5.0CMC, and LDPE without any treatment as control. The developed films were analyzed for their mechanical properties. Subsequently, a storage and application test on *A. bisporus* was performed. Three uniform *A. bisporus* (5.5 ± 0.3 g) selected samples were deposited in a plastic container (LDPE/PVC, volume = 120 mL) and encapsulated with control, P-0.5COL, P-1.0COL, P-3.0COL, P-5.0COL, P-0.5CMC, P-1.0CMC, P- 3.0CMC, and P-5.0CMC, followed by storage at 4 °C for subsequent storage experiments.

### 2.3. Properties of Films

#### 2.3.1. ATR-FTIR Spectra

The ATR–FTIR (Nicolet 6700, Thermo, Waltham, MA, USA) was used to investigate the surface chemical compositions of films treated by plasma, COL, and CMC. The wavenumbers between 4000 and 650 cm^−1^ with 64 scans at a resolution of 4 cm^−1^ were investigated [33].

#### 2.3.2. Film Thickness, Optical Properties, and Mechanical Properties

Film thickness was determined by a Digital Micrometer IP65, item number: 293-330-30 (Mitutoyo, Japan), with a sensitivity of 0.01 mm. Measurements were randomly selected from five different points on the films, and average values were recorded. Opacity measurements were performed according to ASTM D1746-92 [34], the standard test for plastic sheeting, using a UV–VIS spectrophotometer (CT-8600, Chroma Technology, Bellows Falls, VT, USA) to measure the transmission of light at 600 nm. The transparency of films was calculated using the following Equation (1):

(1) $$Opacity=\frac{A_{600}}{x}$$

*A*_600_ is the value of absorbance at 600 nm, and *x* is the film thickness (mm).

#### 2.3.3. Water Vapor, Oxygen, and Carbon Dioxide Permeability of Films

Water vapor permeability (WVP) of films was determined using a slight modification of the previously described ASTM-E96 methodology [35] and using the equation below. Oxygen permeability (OP) and carbon dioxide permeability (CDP) of films were measured by a deoxidizer and alkali absorption methods [14].

### 2.4. Structural Deterioration

#### 2.4.1. Firmness

Firmness (N) of *A. bisporu* was determined by using a TA-XT texture analyzer (TA-XT 2/25, Stable Micro Systems, Godalming, Surrey, England) equipped with a P-35 diameter probe [28].

#### 2.4.2. β-1,3-Glucanase

Extraction was performed with 30 mL of 0.1 M citric acid buffer (pH 5.0) and 0.1 mL of 2 mg/mL dextran solution using 10 g of the sample. DNS was then added and analyzed at an absorbance of 540 nm [36]. 1U is enzyme solution decomposed from β-1,3-glucan solution to release 1 μmol of glucose per minute.

(2) $$\beta -1,3-glucanase=\frac{\left(m\times V\times 10^{3}\right)}{\left(Vs\times t\times M\times 180\right)}$$

where *m* means the glucose content (mg) calculated from the standard curve, *V* means the total volume of the enzyme solution (mL), *Vs* means the volume taken during the determination (mL), *t* means reaction time (min), *M* means sample Weight (g), and 180 is the relative molecular weight of glucose.

#### 2.4.3. Microstructure Evaluation

The center of *A. bisporus* (the 0th and 21st days of the storage period at 4 °C) was obtained, sliced with a thickness of approximately 0.5 mm, and quickly frozen in liquid nitrogen. Freeze-drying was then performed in a freeze dryer for approximately 12 h for obtaining dried *A. bisporus* sample; the conductivity carbon tape was pasted and fixed on a flat stage for surface gilding and observation by scanning electron microscope (JSM-7800F Schottky, JEOL Ltd., Tokyo, Japan) at 1000× magnification.

### 2.5. Oxidative Browning

#### 2.5.1. Browning

A colorimeter was used to measure *L*, *a*, and *b* values of the middle part of the *A. bisporus* cap. The browning index was calculated according to the following Equations (3) and (4):

(3) $$X=\frac{a+1.75L}{\left(5.645L+a-3.012b\right)}$$

(4) $$Browning\;index=\frac{(100\times \left(X-0.31\right)}{0.172}$$

#### 2.5.2. Polyphenol Oxidase

Extraction was performed with phosphate buffer at 0.1 M (pH 6.8) using 10 g of each sample; 0.5 mL of catechol 0.01 M was then added to 5 mL of phosphate buffer solution, and the sample was immediately analyzed at an absorbance of 420 nm. Absorbance value was measured after 2 min. The change in absorbance value per minute is 0.01 as a polyphenol oxidase activity unit (U) and the result is expressed in U kg^−1^ FW [37].

(5) $$Polyphenol\;oxidase\;activity=\frac{\Delta OD_{420}\times V}{0.01\times Vs\times t\times m}$$

where *V* means total volume of enzyme solution (mL), *Vs* means volume taken during determination (mL), *t* means reaction time (min), and m means fresh weight of sample (kg).

### 2.6. Respiratory Injury

#### 2.6.1. Electrolyte Leakage

*A. bisporus* (2.0 g) was weighed, the dirty surface was washed with distilled water, and the excess water on the surface was wiped. They were then watered, cut into pieces, and placed in 50 mL of distilled water at 25 °C for 3 h. Conductivity (*C*1) was then measured using a conductivity meter, and the sample was boiled for 10 min and cooled to 25 °C to test the final conductivity (*C*2) [37]. The relative conductivity was calculated using Equation (6) as follows:

(6) $$Electrolyte\;leakage\;rate=\left(\frac{C1}{C2}\right)\times 100\%$$

#### 2.6.2. Malondialdehyde

Extraction was performed with 10 mL 10% (*v*/*v*) trichloroacetic acid (TCA) using 10 g of each sample; 2 mL of 0.6% (*v*/*v*) thiobarbituric acid (TBA) solution (prepared with 10% TCA) was then added, and samples were analyzed at absorbance of 532, 600 and 450 nm [38].

(7) $$MDA\;\left(\frac{\mu mol}{kg}\right)=6.45\left(OD_{532}-OD_{600}\right)-0.56\times OD_{450}$$

#### 2.6.3. Catalase

Extraction was performed with 50 mL of phosphate buffer at 0.05 M (pH 7.8) using 10 g of each sample; 1.7 mL of 0.1 M phosphate buffer solution (pH 7.8) and 1 mL of 0.1 M H_2_O_2_ solution were then added. Samples were analyzed at 240 nm, and the absorbance values were recorded every 30 s until 3 min, corresponding to one catalase activity unit (U). The result was expressed in Ukg^−^^1^ FW [39].

(8) $$Catalase\;activity=\frac{\Delta OD240\times V}{0.01\times Vs\times t\times m}$$

where *V* means total volume of enzyme solution (mL), *Vs* means volume taken during determination (mL), *t* means reaction time (min), and m means fresh weight of sample (kg).

#### 2.6.4. Respiration Rate

Every three days, the CO_2_ and O_2_ concentrations in different packages containing *A. bisporus* were measured using a gas analyzer (PA7.0 version S, Witt-Gasetechnik, Witten, Germany). Air was used as calibration before each measurement. The gas composition of each package was measured in triplicate, and the results were reported as expected percentages of air composition (%) [14].

### 2.7. Data Analysis

Data were expressed as mean ± standard deviation. All experiments were performed with triplicate. All data were analyzed with SPSS (version 20.0, SPSS Inc, Chicago, IL, USA). Data were subjected to analysis of variance, and mean comparison was performed by Duncan’s multiple comparisons tests. Differences were considered significant at *p*-values of <0.05 [40].

## 3. Results and Discussion

### 3.1. Properties of Different Packaging

**Figure 1:**
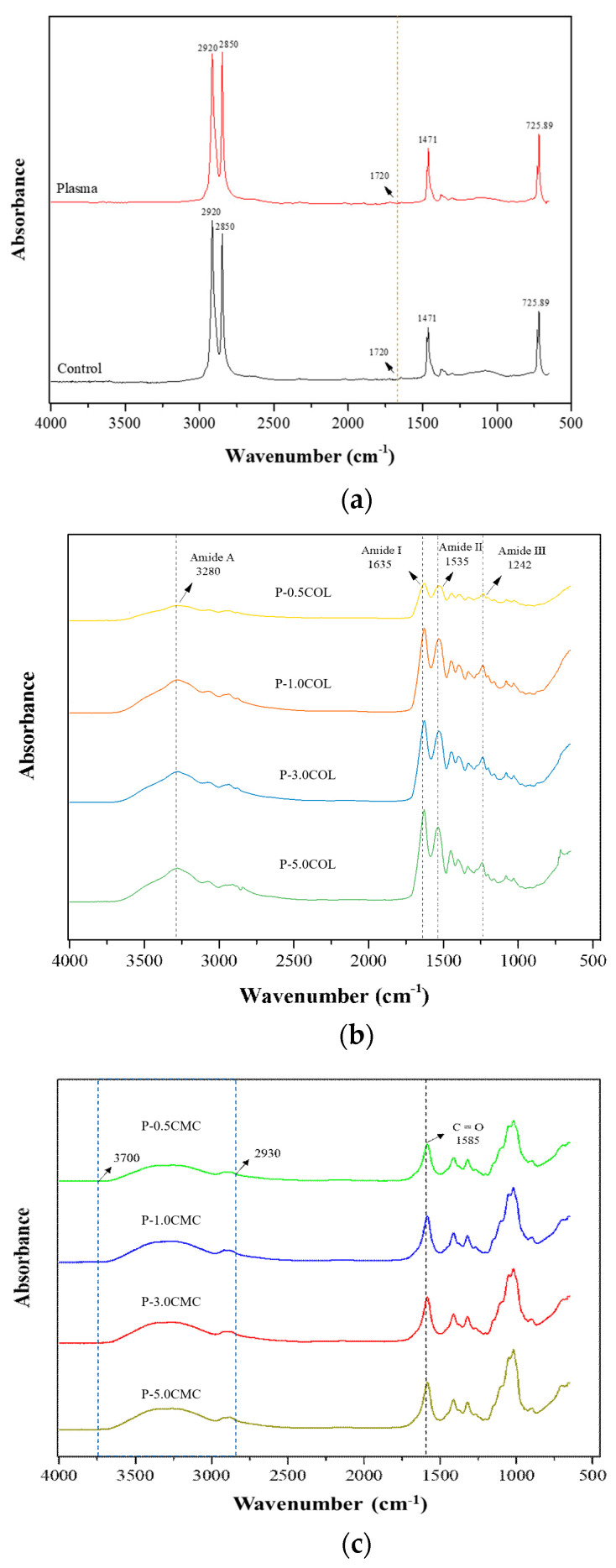
Surface functional groups of different films: (**a**) control, (**b**) COL group, and (**c**) CMC group.

On the basis of the spectral bands of the ATR-FTIR scan of the film in Figure 1, we found that the control group (LDPE film) was mostly composed of methylene (CH_2_) and that there were four main peaks of methylene extension that corresponded to 2920 and 2850 cm^−1^. At 2856, 1471, 1162, and 725.89 cm^−1^, the bending vibration corresponded to methylene. After plasma treatment, a new peak was found at 1720 cm^−1^, which corresponded to C=O stretching vibration [33], as shown in Figure 1a.

**Figure 2:**
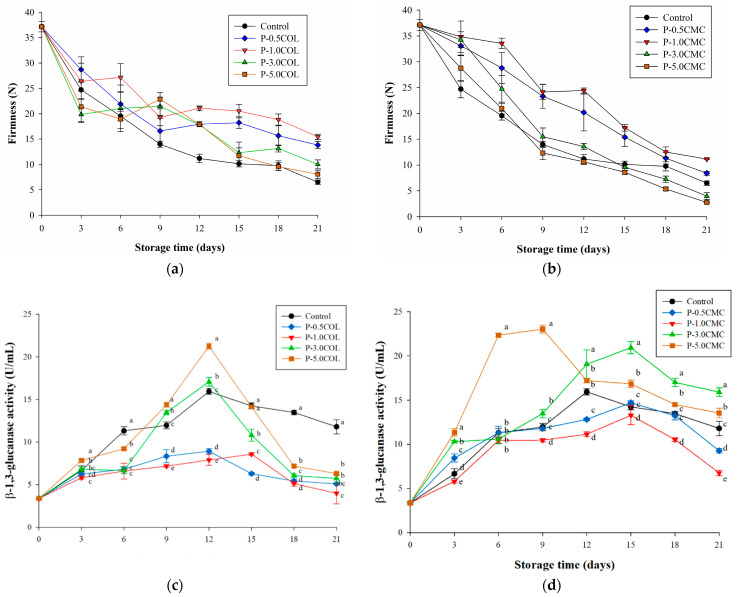
Effects of different packaging films on the structure of *A. bisporus* during storage at 4 °C for 21 days. (**a**) Firmness decline in different COL packaging films. (**b**) Firmness decline in different CMC packaging films. (**c**) β-1,3-Glucanase activity changed in different COL packaging films. (**d**) β-1,3-Glucanase activity changed in different CMC packaging films. Vertical bars represent the standard deviations of five replicates. ^a–e^ Means followed by different superscripts indicate the statistically significant different values among treatments for the same storage time (*p* < 0.05).

In Figure 2b, the collagen films coated with different concentrations corresponded to the stretching vibration of the amide bond (Amide A) at 3280 cm^−1^ and the stretching and C=O bond at 1635 cm^−1^. NH bonding corresponded to Amide I, whereas 1535 cm^−1^ and 1242 cm^−1^ corresponded to the tensile vibration of Amide II and Amide III, which were characteristic peaks of collagen, and this could have also been observed in previous studies [41]. Figure 1c also shows a film coated with different concentrations of carboxymethyl cellulose, where 1585 cm^−1^ corresponds to the stretching of asymmetric C=O bond carboxylate group (COO-) [42] and 2900–3700 cm^−1^ corresponds to the stretching of the hydroxyl groups in carboxymethyl cellulose [41]. In related studies, it is also highlighted that the hydroxyl groups of CMC and the amide groups of COL have the ability to select carbon dioxide [20]; therefore, the results of this part show that the modification of COL and CMC can indeed provide the LDPE surface a functional group with gas-selective properties.

Table 1 summarizes the film properties of different polymer materials coated with plasma-treated LDPE. In transparency analysis, there was no significant difference among P-0.5COL, P-0.5CMC, and control. However, when concentrations of COL and CMC increased (from 1.0 to 5.0%), a slight impact on the transparency of the film was observed, especially for the CMC group, in which the impact was more significant. The thickness analysis also showed that CMC modification resulted in a significant change in the thickness of the film. The thickness of P-5.0MC was approximately twice of the control. Although COL group modification also affected film thickness, the effect was not as significant as that of CMC, suggesting that this is the main factor affecting light transmittance.

It can be observed in Table 1 that compared with the control group, the tensile strength of COL and CMC groups increased with concentration, but when concentration was increased to 5.0%, the tensile strength of the polymers coated with CMC and COL significantly decreased. Related research showed that as the concentration of natural polymer colloids such as COL or CMC increased, the tensile strength of the film decreased [43]. Consequently, when the concentration of COL or CMC was higher, a brittle and poorly ductile film would form on the surface of LDPE, owing to polymer gelation, resulting in a decrease in the overall mechanical strength, especially tensile strength [17,43].

Table 1 shows that the addition of CMC 0.5% and 1.0% had a marginal effect on the improvement of the WVP of LDPE by <0.1, whereas the addition of COL at 0.5% and 1% significantly increased the WVP of LDPE by 2–3 times. However, when the amount of COL and CMC was increased to >3.0%, the WVP of LDPE was not only extremely increased but a condensation on the inside and outside of the packaging film was also observed, which indicated that modification of the samples COL and CMC at a concentration of at least 3.0% can lead to easy absorption of water and can adversely affect the discharge of water vapor. Excessive moisture accumulated in the packaging will adversely affect the preservation of agricultural products [44].

Table 1 shows that the control group had higher OP and CDP, as well as the fact that the addition of CMC and COL can significantly reduce the permeability of OP in a similar manner, partly because the polymer material coated on LDPE may form a complex double-layer film structure, which further reduces the OP [19]. Conversely, there is a significant difference in the reduction of CDP. Although both CMC and COL reduce CDP, reduction of COL group is greater than that of the CMC group. A possible cause for this phenomenon as shown in previous studies is that polymer materials with amine-based structures have a higher adsorption effect on carbon dioxide [45]. These results clearly show that when CMC and COL were coated on the plasma-treated LDPE, a small effect on the tensile strength and transmittance of the LDPE was observed, which could cause a change in gas permeability.

### 3.2. Effects of Different Packaging on the Structure in A. bisporus during Storage

After harvesting, vigorous respiration will cause β-glucanase to accelerate the decomposition of the cell wall’s polymer structure into monosaccharides. Consequently, the structure of the mushroom body changes from moderately soft and flexible to soft and rotten, and it is not possible to maintain the normal shape of the mushroom body [46].

Figure 2a,b presents the firmness decline of *A. bisporus* after storage at 4 °C for 21 days. Relevant literature shows that the firmness of *A. bisporus* gradually decreases with time after storage, and this may be due to shrinkage of the mycelium and destruction of the central vacuole [47]. In this experiment, the firmness of the control group decreased from 37.14 N at the beginning of storage to 6.47 N after 21 days of storage, and the firmness was reduced by approximately 85%. The firmness decrease of 0.5% CMC and COL group was between 70% and 80%. Only 1.0% COL and CMC group had <70% of firmness decrease, with 1.0% COL having the smallest decrease (61.09%). The P-0.5COL and P-0.5CMC groups probably contain relatively low O_2_ and higher CO_2_, owing to the strong gas barrier properties of the package. This would lead to anaerobic respiration, and the CO_2_ stress would make the maintenance of the firmness of *A. bisporus* more difficult. Previous research [48] investigated the effect of combing modified atmosphere packaging with a bilayer active film on the shelf life of oyster mushroom. In that study, *A. bisporus* packaged with high oxygen atmosphere showed a firmness decline rate for about 96.93% after storage. The coating effect, however, at 5% of CMC and COL was similar to that of the control, possibly due to the fact that O_2_/CO_2_ permeability ratio was similar to that of the control.

Figure 2c presents the β-1,3-glucanase activity of P-3.0COL and P-5.0COL on the 12th day, owing to intense β-1,3-glucanase activity, which was higher than the control when stored for 21 days. On the contrary, P-1.0COL and P-.05COL maintained lower β-1,3-glucanase activity (6 and 8 U/mL, separately at the 12th day), suggesting that they showed an improved firmness maintenance effect. In the CMC group, P-5.0CMC (24 U/mL) had a significant increase in β-1,3-glucanase activity on the 3rd day, whereas P-3.0CMC (13 U/mL) showed increase on the 9th day Figure 2d. Nevertheless, the activity of P-5.0CMC began to decline on the 9th day and the activity of P-3.0CMC did not show a decreasing trend until the 15th day. The β-1,3-glucanase activity of both P-3.0CMC (17 U/mL) and P-5.0CMC (15 U/mL) were still higher than that of the control (13 U/mL) group on day 21, resulting in the same as P-3.0COL and P-5.0COL. P-1.0CMC and P-0.5CMC also showed improved firmness maintenance effect as they maintained a lower β-1,3-glucanase activity (13 and 15 U/mL, respectively) on the 15th day. Because β-glucan and chitin are connected by glycosidic bonds in their main structure [49], P-1.0CMC, P-0.5CMC, P-1.0COL, and P-0.05COL can effectively maintain *A. bisporus* structure by inhibiting β-1,3-glucanase activity.

Table 2 shows that P-1.0COL and P-1.0CMC retained a structure similar to that of fresh samples (control, day 0) with complete water retention, elasticity, and toughness after being stored for 21 days. In P-0.5COL, P-0.5CMC, P-3.0COL, P-5.0COL, P-3.0CMC, and P-5.0CMC, network structure loss and damage can be observed. It was also confirmed that P-1.0COL and P-1.0CMC can maintain structure by regulating β-1,3-glucanase. Conversely, the other treatment groups were unable to effectively inhibit the activity of β-1,3-glucanase, thereby resulting in failure in the effective reduction of the tissue structure collapse.

Previous studies have demonstrated that the use of micro-perforation air-conditioning packaging promotes anaerobic respiration of *A. bisporus* owing to gas saturation after the 10th day, which may consequently lead to the disintegration of the structure [8,10]. In P-3.0CMC, P-5.0CMC, P-3.0COL, and P-5.0COL packaging films, owing to low O_2_ permeability with high CO_2_ permeability, CO_2_ was easier to be removed from the package.

Respiration of *A. bisporus* in the package continued at the same time to promote respiration, resulting in an increase in the β-1,3-glucanase activity. Furthermore, 1.0% COL- and CMC-modified packaging may offer more effective inhibition of respiration and thus an improved inhibitory effect on β-1,3-glucanase.

### 3.3. Effects of Different Packaging on Browning in A. bisporus during Storage

**Figure 3:**
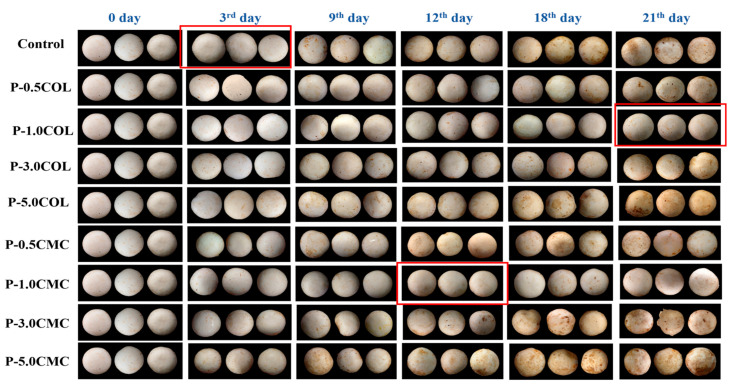
Appearance of *A. bisporus* after different packages stored at 4 °C for 21 days.

The browning index of *A. bisporus* was increased with storage time. The appearance and degree of browning of the product are the key factors determining consumer’s purchasing intention. Figure 3 shows that on the 9th day, the cap of *A. bisporus* packaged in P-3.0COL, P-3.0CMC, P-5.0COL, and P-5.0CMC groups showed significant brown spots. This phenomenon increased with the storage time, and its severity also increased until the 21st day. P-0.5CMC showed significant browning on the 18th day, whereas P-0.5COL did not show significant browning until the 21st day. However, P-1.0CMC and P-1.0COL maintained good appearance until the 21st day.

**Figure 4:**
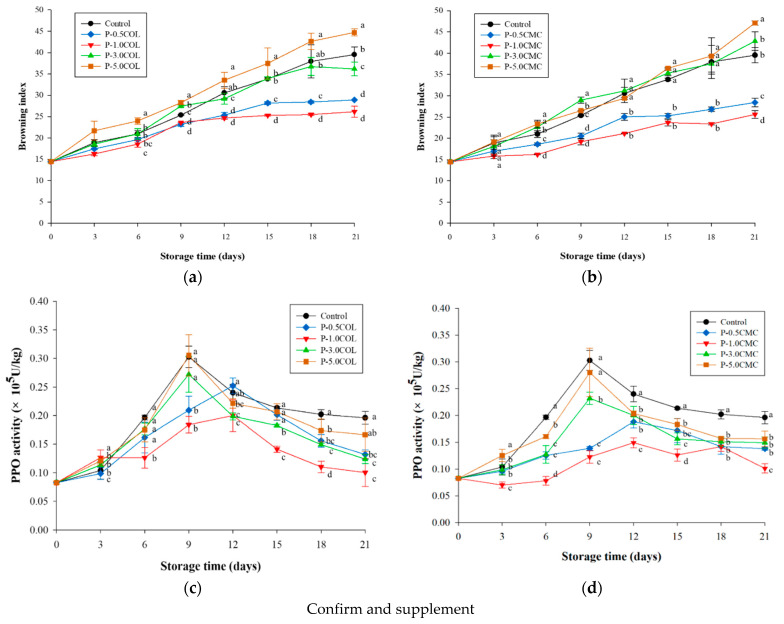
The effects of different packaging films in the oxidative browning of *A. bisporus* stored at 4 °C for 21 days. (**a**) Browning index in different COL packaging films. (**b**) Browning index in different CMC packaging films. (**c**) PPO activity changes in different COL packaging films. (**d**) PPO activity changes in different CMC packaging films. Vertical bars represent the standard deviations of five replicates. ^a–e^ Means followed by different superscripts indicate the statistically significant different value among treatments at the same storage time (*p* < 0.05).

Figure 4a,b shows that the browning index of P-0.5CMC, P-1.0CMC, P-0.5COL, and P-1.0COL was still below 30%, whereas it was approximately 25% for all after 21 days. The browning of control, P-3.0COL, P-5.0COL, P-3.0CMC, and P-5.0CMC exceeded 30% on the 21st day, which meant 3% and 5% of CMC. COL cannot provide more effective quality maintenance for *A. bisporus*. Relevant literature pointed out that when the browning degree of Pleurotus eryngii is greater than 25%, consumers’ purchasing intention is significantly reduced [48]. The results of this stage showed that P-0.5CMC, P-1.0CMC, P-0.5COL, and P-1.0COL can maintain *A. bisporus* appearance after 21 days of storage; it can still maintain a certain degree of consumer willingness to purchase.

In Figure 3, control, P-3.0COL, P-3.0CMC, P-5.0COL, and P-5.0CMC groups reached the highest peak of PPO activity (0.3, 0.27, 0.22, 0.3, 0.28 U/kg) on the 9th day of storage. For P-0.5COL, P-0.5CMC, P-1.0COL, and P-1.0CMC, the highest peak of PPO activity (0.25, 0.18, 0.19, and 0.14 U/kg) was not observed before the 12th day. In addition, P-1.0 COL and P-1.0CMC can effectively inhibit PPO activity by approximately 50% during storage compared with the control group. In previous studies, the use of chemical preservatives could only inhibit approximately 42% of PPO activity of *A. bisporus* [1], showing that the active packaging film in this study can effectively inhibit the activity of PPO by delaying the respiration of *A. bisporus* and maintaining the mushrooms integrity. The content of phenolic compounds further maintains the appearance, degree of browning, and nutritional value of *A. bisporus* during storage

Previous studies used MAP or micro-perforation packaging to preserve the freshness of *A. bisporus*, with most of them showing significant browning from the 14th day [16]. Related research also showed that when the browning degree of *Pleurotus ostreatus* exceeds 25, consumers’ purchasing intention is greatly reduced [50]. The results of the current study show that the browning degree of P-1.0COL and P-1.0CMC was reduced on the 21st day (less than 25) combined with improved appearance, which is extended by at least 12 days compared with the control group.

### 3.4. Effects of Different Packaging on Cell Membrane Damage in A. bisporus during Storage

**Figure 5:**
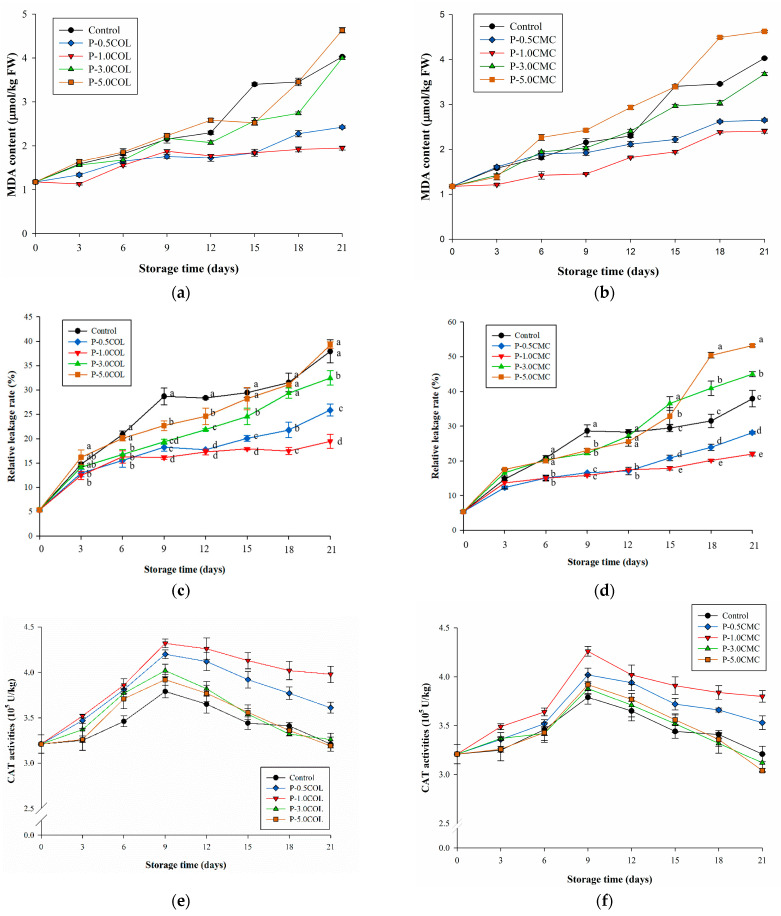
Effects of different packaging films on respiratory injury of *A. bisporus* stored at 4 °C for 21 days. (**a**) MDA in different packaging in different COL packaging films. (**b**) MDA in different packaging in different CMC packaging films. (**c**) Relative leakage in different COL packaging films. (**d**) Relative leakage in different CMC packaging films. (**e**) CAT activity changed in different COL packaging films. (**f**) CAT activity changed in different CMC packaging films. ^a–e^ Means followed by different superscripts indicate the statistically significant different values among treatments at the same storage time (*p* < 0.05).

Figure 5 demonstrates the evaluation of the resistance to cell membrane damage of LDPE coated with different COL and CMC ratios. Figure 5a,b shows that the increase in MDA of P-0.5COL (2.4 μmol/kg FW), P-1.0COL (1.9 μmol/kg FW), P-0.5CMC (2.6 μmol/kg FW), and P-1.0CMC (2.4 μmol/kg FW) was lower than that of the control group (4.0 μmol/kg FW) during storage. Conversely, P-3.0COL (3.9 μmol/kg FW), P-5.0COL (4.6 μmol/kg FW), P-3.0CMC (3.6 μmol/kg FW), and P-5.0CMC (4.6 μmol/kg FW) were not significantly different from the control group during storage. This may be owing to the higher CDPs of P-3.0CMC, P-5.0CMC, P-3.0COL, and P-5.0COL, which promotes oxygen exchange rate inside and outside the package, leading to a more active respiration of *A. bisporus* in the packaging and promotion of oxidative damage occurrence in cell membrane [51].

Figure 5c,d shows that P1.0-COL and P1.0-CMC were able to inhibit the increase in relative leakage rate (relative leakage rates were approximately 20% and 22%, respectively). P-3.0COL, P-3.0CMC, P-5.0COL and P-5.0CMC inhibited the increase in relative leakage rate before the 12th day, but the relative leakage rate of the four groups then was exceeded to 22% in all groups. In addition, P-3.0CMC and P-5.0CMC caused a more significant relative leakage, similar to MDA, which may have been caused by excessive respiration. Moreover, although P-0.5CMC and P-0.5COL can reduce cell membrane damage by almost 15% by reducing OP (compared with control group), they were less efficient compared with P-1.0CMC and P-1.0COL (20%). Moreover, the effect showed that lower gas barrier was effective in inhibiting respiration, but moderate O_2_ and CO_2_ permeability can reduce the oxidative damage of the cell membrane of *A. bisporus* more effectively.

Previous research [52] pointed out that when the oxygen content in the packaging was appropriate, it could increase the oxidative stress of the cells, thus inducing CAT activity, which is beneficial in delaying the oxidation process of *A. bisporus* and maintaining its quality.

Figure 5e,f further confirms that the CAT activity of *A. bisporus* can be maintained by plasma modification and adjustment of different polymers. The trends of P-3.0COL, P-5.0COL, P-3.0CMC, P-5.0CMC, and control were similar. On the ninth day, the CAT activity of P-3.0COL, P-5.0COL, P-3.0CMC, P-5.0CMC, and control was between 3.6 and 3.7 × 10^5^ U/kg, and then there were declines below 3.5 × 10^5^ U/kg on 21th day. The CAT activity of P-0.5COL, P-1.0COL, and P-1.0CMC increased to more than 4 × 10^5^ U/kg on the 9th day, and the CAT activity of P-0.5CMC was about 3.9 × 10^5^ U/kg. After that, the CAT activities of P-0.5COL, P-1.0COL, P-0.5CMC, and P-1.0CMC were decreasing but still maintained above 3.5 × 10^5^ U/kg, and P-1.0COL was even maintained at about 4 × 10^5^ U/kg. Figure 4e,f also demonstrates that the use of P-1.0COL can effectively maintain CAT activity and thus inhibit the rupture of cell membrane caused by its lipid oxidation and subsequent quality deterioration.

### 3.5. Effects of Different Packaging on the Respiration in A. bisporus during Storage

**Figure 6:**
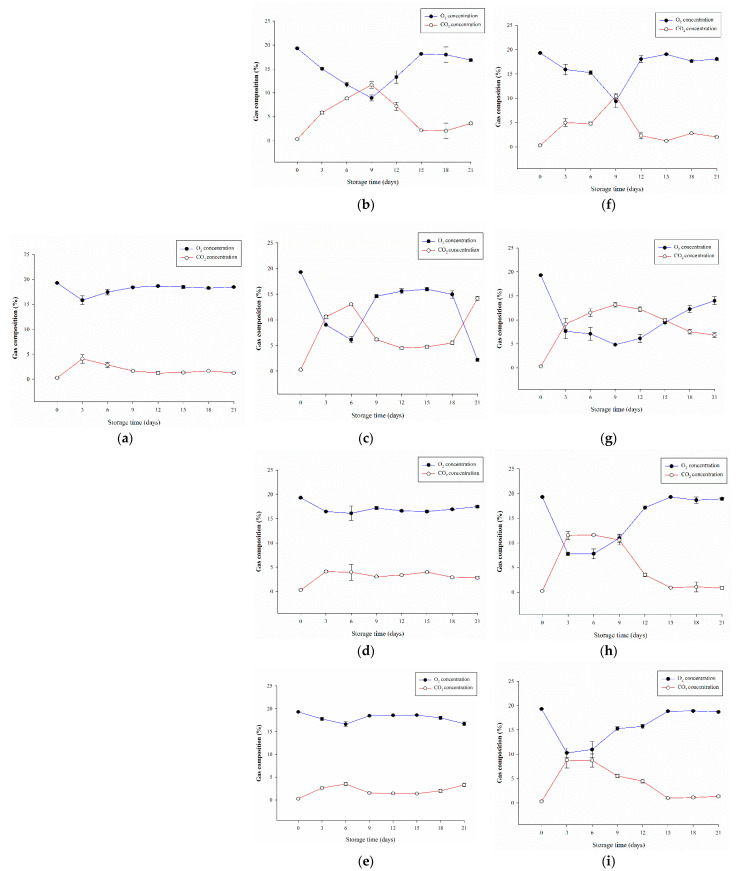
The effects of different packaging films on respiration of *A. bisporus* stored at 4 °C after 21 days. (**a**) Control. (**b**) P-0.5CMC. (**c**) P-1.0CMC. (**d**) P-3.0CMC. (**e**) P-5.0CMC. (**f**) P-0.5COL. (**g**) P-1.0COL. (**h**) P-3.0COL. (**i**) P-5.0COL. ^a–e^ Means followed by different superscripts indicate the statistically significant different values among treatments at the same storage time (*p* < 0.05).

Figure 6 shows the capacity of different films in regulating the gas composition in the packaging during storage. As per our results, compared with the control with LDPE packaging, P-3.0CMC and P-5.0CMC exhibited a similar trend. During the 21-day storage period, there was no way to control the gas composition in the package. Most of the O_2_ content was >18%, and the CO_2_ content was mostly between 1% and 2%, similar to the general atmospheric composition. Therefore, the preservation effects of control, P-3.0CMC, and P-5.0CMC were mostly similar and could not produce effective preservation effects.

Although P-0.5CMC can effectively reduce the O_2_ content to approximately 9.0% in the first 9 days, it will still revert to a high-oxygen environment by >15% after the 9th day and cannot maintain the gas composition in the package. P-1.0CMC can reduce the O_2_ content in the package to <10% in the first 3 days, but still cannot maintain the suppression of the increase in O_2_ concentration after the 9th day. Relevant studies have pointed out that the respiration of *A. bisporus* during cold storage will occur from the 3rd to the 6th days [1,2]; P-1.0CMC may exhibit a better preservation effect by inhibiting the exuberant respiration process.

The trend of P-0.5COL is similar to that of P-0.5CMC. Although it can suppress the O_2_ content to approximately 10% before the 9th day, it cannot maintain the effect of regulating the gas composition in the package. P-1.0COL can suppress O_2_ content to <10% and >5% within 3 days and CO_2_ content to >10% and <15% after packaging and maintain these for 15 days. Afterwards, although the O_2_ content gradually increased, and the O_2_ content was 15%–16% on the 21st day, which was still far below the 18%–19% of the general atmospheric composition, showing that 1.0COL combined with plasma modification can develop an EMAP. P-3.0COL is similar to P-1.0CMC. Although it could effectively suppress the O_2_ content in the initial stage, it could not effectively suppress the increase in O_2_ concentration after the 9th day; therefore, the storage effect was slightly inferior to P-1.0CM. P-5.0COL was similar to the control, and could not effectively control the gas composition.

Related research pointed out that although both CMC and COL have gas selection characteristics [19,20,21], the results of this research stage showed that the O_2_ suppression effect of COL was better than that of CMC. This may have been because the functional group of COL is more selective for O_2_ and CO_2_ than that of CMC. Therefore, the gas barrier and the subsequent gas maintenance in the packaging were more significant in the COL group. This study also shows that in the future, the combination of plasma modification and appropriate polymers and their concentrations should be able to develop EMAP suitable for various agricultural products with unique respiration characteristics.

## 4. Conclusions

The results of the current study confirm that the use of plasma modification with different concentrations of CMC and COL allowed for the control of gas selectivity of LDPE without affecting its mechanical properties. Application preservation experiments also confirmed that both P-1.0CMC and P-1.0COL were able to inhibit the activity of PPO and β-1,3-glucanase to achieve delayed tissue decomposition and oxidative browning. Furthermore, P-1.0CMC and P-1.0COL can resist the oxidative damage of a cell membrane by maintaining the activity of catalase. However, it can be known from the analysis of respiration that P-1.0COL can more effectively maintain the oxygen (<10% and >5%) composition in the package during the storage period (21 days). Therefore, P-1.0COL is more effective in maintaining the composition of CO_2_ and O_2_ (carbon dioxide: 10%–15% and oxygen: 8%–15%) during storage. It has a more stable performance in maintaining the tissue structure and oxidizing browning of *A. bisporus*. It has also been proved that the combination of plasma technology and natural polymer materials lead to the development of a preservation packaging material suitable for high-respiration agricultural products from *A. bisporus* with an extended shelf life from 7 days to 21 days.

## Figures and Tables

**Table 1:** Mechanical properties of different films.

	Thickness (mm)	Transparency	Tensile strength (MPa)	WVP (×10^–7^ g∙mm∙m^−2^∙h^−1^∙Pa^−1^)	OP (×10^–12^ g∙m∙m^−2^∙s^−1^∙Pa^−1^)	CDP (×10^–12^ g∙m∙m^−2^∙s^−1^∙Pa^−1^)
Control	0.033 ± 0.001 ^f^	1.500 ± 0.021 ^e^	19.25 ± 0.10 ^f^	0.29 ± 0.10 ^d^	4.18 ± 0.24 ^a^	8.98 ± 0.48 ^a^
P-0.5COL	0.034 ± 0.001 ^f^	1.508 ± 0.079 ^e^	24.52 ± 0.67 ^c^	0.61 ± 0.31 ^cd^	2.42 ± 0.14 ^d^	2.42 ± 1.45 ^d^
P-1.0COL	0.037 ± 0.002 ^e^	1.614 ± 0.031 ^de^	28.38 ± 0.30 ^a^	0.79 ± 0.28 ^cd^	2.38 ± 0.12 ^d^	3.17 ± 0.03 ^d^
P-3.0COL	0.045 ± 0.002 ^d^	1.662 ± 0.072 ^de^	25.99 ± 0.28 ^b^	1.61 ± 0.14 ^c^	2.40 ± 0.09 ^d^	3.64 ± 0.59 ^d^
P-5.0COL	0.059 ± 0.002 ^c^	1.659 ± 0.066 ^de^	21.17 ± 0.26 ^e^	8.63 ± 1.35 ^a^	2.67 ± 0.22 ^c^	5.88 ± 0.06 ^bc^
P-0.5CMC	0.036 ± 0.002 ^ef^	1.523 ± 0.006 ^e^	26.23 ± 1.19 ^b^	0.36 ± 0.12 ^cd^	2.69 ± 0.11 ^c^	2.67 ± 0.11 ^d^
P-1.0CMC	0.043 ± 0.002 ^d^	1.733 ± 0.011 ^c^	29.30 ± 0.55 ^a^	0.37 ± 0.25 ^cd^	2.77 ± 0.23 ^b^	3.54 ± 0.28 ^d^
P-3.0CMC	0.065 ± 0.001 ^b^	1.947 ± 0.069 ^b^	22.42 ± 0.93 ^d^	1.05 ± 0.18 ^cd^	2.80 ± 0.30 ^b^	5.63 ± 0.30 ^c^
P-5.0CMC	0.069 ± 0.002 ^a^	2.591 ± 0.325 ^a^	15.34 ± 0.24 ^g^	4.57 ± 1.51 ^b^	3.87 ± 0.56 ^a^	6.85 ± 0.49 ^b^

Values are presented as the mean ± standard deviation (n = 5). ^a–f^ Means followed by different superscripts in the same column are significantly different (*p* < 0.05).

**Table 2:** Effects of different packaging on the microstructure (×1000) in *A. bisporus* after storage at 4 °C for 21 days.

Day 0	Day 21				
Control	Control	P-0.5COL	P-1.0COL	P-3.0COL	P-5.0COL
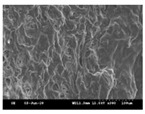	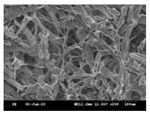	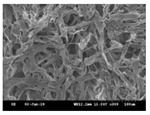	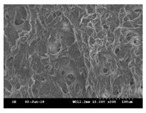	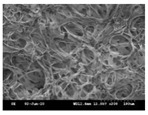	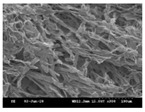
		P-0.5CMC	P-1.0CMC	P-3.0CMC	P-5.0CMC